# Supplementary material for: Pharmacologically induced amnesia for learned fear is time and sleep dependent
Source: Nat Commun. 2018 Apr 3;9:1316. doi: 10.1038/s41467-018-03659-1 (PMC5883008; doi:10.1038/s41467-018-03659-1)
Supplement: Supplementary file 1 — Supplementary Information(PDF 745 kb) [file 41467_2018_3659_MOESM1_ESM.pdf]

Pharmacologically induced amnesia for learned fear is time and sleep-dependent

Supplementary Information

Merel Kindt [1,2], Marieke Soeter[1,3]

[1] University of Amsterdam, Department of Clinical Psychology, Nieuwe Achtergracht 129, 1018 WS, Amsterdam, The Netherlands.

[2] Amsterdam Brain and Cognition Center, Amsterdam, The Netherlands.

[3] TNO, Microbiology and Systems Biology, Zeist, The Netherlands.

	experiment_1		experiment_2		experiment_3	
	propranolol	nadolol	pill_1h	pill_2h	no_sleep	sleep
group sizes*	n = 15, 6 men	n = 15, 7 men	n = 10, 5 men	n = 10, 4 men	n = 10, 7 men	n = 10, 5 men
age	20.9 ± 2.3	21.5 ± 3.0	25.6 ± 8.0	21.9 ± 2.4	21.7 ± 3.8	23.8 ± 5.4
STAI	34.7 ± 10.0	30.0 ± 5.2	33.9 ± 8.6	34.7 ± 8.0	32.3 ± 9.5	31.0 ± 7.1
ASI	10.4 ± 4.4	8.3 ± 3.8	9.0 ± 5.5	8.9 ± 5.4	8.6 ± 5.6	10.0 ± 5.6
FSQ	27.2 ± 16.5	27.9 ± 13.6	31.3 ± 23.8	28.8 ± 20.1	22.7 ± 4.5	31.5 ± 17.81
US evaluation	3.5 ± 0.6	3.5 ± 0.9	3.3 ± 0.8	3.3 ± 1.1	3.0 ± 0.7	2.7 ± 0.5
US mA	17.8 ± 9.9	15.7 ± 8.3	14.1 ± 6.9	19.4 ± 12.5	15.6 ± 7.2	15.0 ± 11.8

Supplementary table 1 | Participant characteristics. Mean values ± SD of age, trait anxiety[6], anxiety sensitivity[7] and reported spider fear[8] as well as US intensity and US evaluation, ranging from 0 to 5 and where higher is more aversive. In all three experiments the participants were randomly assigned to one of two groups. All participants were assessed to be free from any current or previous medical or psychiatric condition that would contraindicate taking a single β-blocker of 40 mg: i.e., pregnancy, seizure disorder, respiratory disorder, cardiovascular diseases, BP < 90/60, liver and kidney disorders, current depression or psychosis. When a participant was medically cleared the STAI-T[6], ASI[7] and FSQ[8] were administered. Participants received either experimental-credits or a small amount of €50 for their participation in the study. Informed consent was obtained from all participants and the ethical board of the University of Amsterdam approved the study. Note that comparisons between groups on the above described variables did not reveal any differences: experiment_1, $ts_{28} < 1.43$; experiment_2, $ts_{18} < 1.40$; and experiment_3, $ts_{18} < 1.51$.

*Present sample sizes are too small to properly test for gender effects. But it is worth noting that in our previous studies we have never detected any differences between the gender groups.

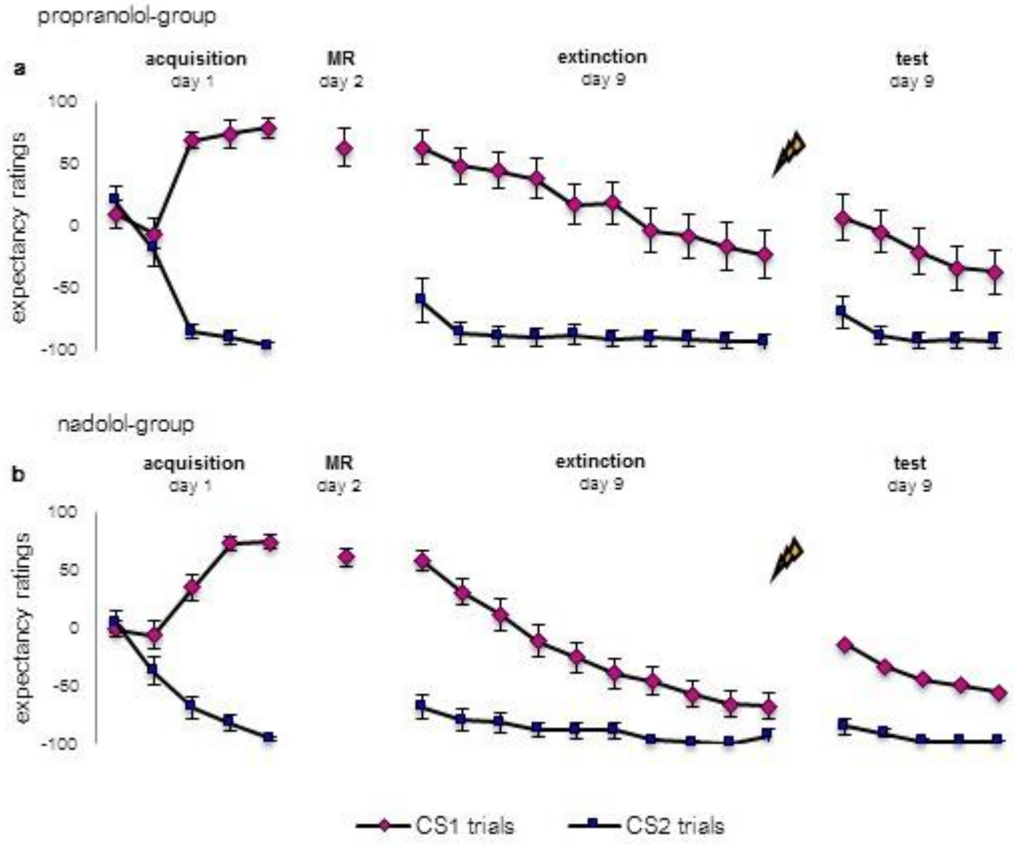

Supplementary Figure 1 | US expectancy ratings are not affected by both β-adrenergic manipulations. Mean US expectancies to the CS1 and CS2 trials during acquisition as well as reactivation and test for **a.** the propranolol-group and **b.** the nadolol-group. MR refers to memory reactivation. Error bars represent s.e.m. US expectancy ratings did not differ between groups [CS1 *vs.* CS2 - stimulus x trial x group - $Fs_{1,28} < 2.47$, $Ps > 0.127$]. In both groups the US expectancy ratings increased during fear acquisition [CS1 *vs.* CS2 - stimulus x trial - $F_{1,28}= 202.67$, $P < 0.001$, $\eta_p^2 = .88$] and all participants were aware of the CS-US contingencies immediately after they underwent the conditioning procedure. Moreover, the expectancy ratings decreased from the last acquisition trial to the first trial of extinction training [CS1 *vs.* CS2 - stimulus x trial - $F_{1,28} = 13.52$, $P = 0.001$, $\eta_p^2 = .33$], indicating a prediction error (PE) upon memory reactivation, which is a necessary condition for the reconsolidation of associative fear memory[1,2]. A further reduction in US expectancy was observed during extinction learning [CS1 *vs.* CS2 - stimulus x trial - $F_{1,28} = 28.36$, $P < 0.001$, $\eta_p^2 = .50$]. However, the reminder shocks did not result in a significant differential return of the US expectancies in both groups [CS1 *vs.* CS2 - stimulus x trial $F < 1.43$, $P = 0.241$].

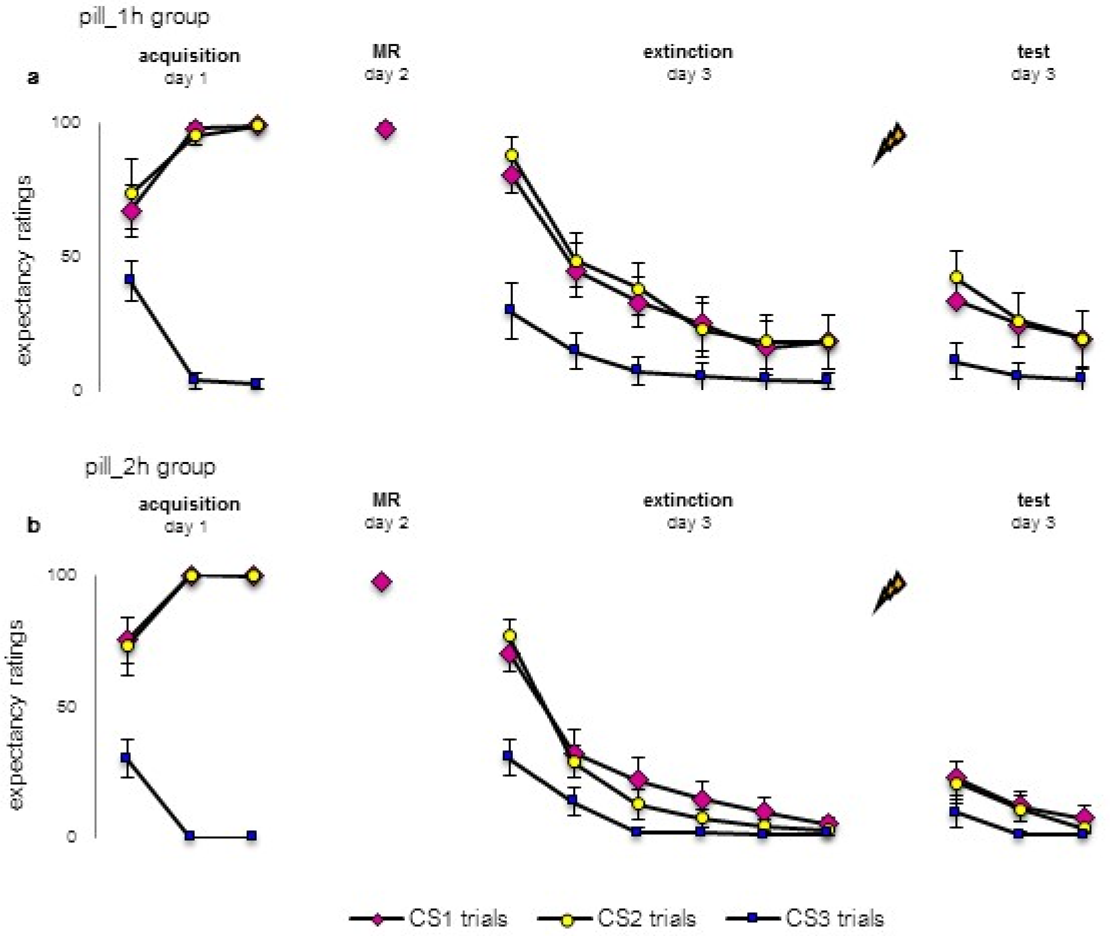

Supplementary Figure 2 | Propranolol does not affect the US expectancy ratings. Mean US expectancies to the CS1, CS2 and CS3 trials during acquisition as well as reactivation and test for **a.** the pill_1h group and **b.** the pill_2h group. MR refers to memory reactivation. Error bars represent s.e.m. Again the US expectancy ratings did not differ between groups [i.e., simple contrasts: CS1 *vs.* CS3 and CS2 *vs.* CS3 - stimulus x trial x group - $Fs_{1,18} < 1.30$, $Ps > 0.270$]. In both the pill_1h and pill_2h group the US expectancies increased during fear acquisition [CS1 *vs.* CS3 and CS2 *vs.* CS3 - stimulus x trial - $F_{1,18} = 58.87$, $P < 0.001$, $\eta_p^2 = .77$ and $F_{1,18} = 34.19$, $P < 0.001$, $\eta_p^2 = .65$] and all twenty participants were aware of the stimulus contingencies. Furthermore, the expectancy ratings decreased from the last acquisition trial to the first extinction trial [CS1 *vs.* CS3 and CS2 *vs.* CS3 - stimulus x trial - $F_{1,18} = 60.69$, $P < 0.001$, $\eta_p^2 = .77$ and $F_{1,18} = 49.26$, $P < 0.001$, $\eta_p^2 = .73$], again demonstrating PE driven learning[1,2]. US expectancy ratings further decreased over the course of the extinction learning process [CS1 *vs.* CS3 and CS2 *vs.* CS3 - stimulus x trial - $F_{1,18} = 11.52$, $P < 0.01$, $\eta_p^2 = .39$ and $F_{1,18} = 17.50$, $P = 0.001$, $\eta_p^2 = .49$] and in both groups we observed a return in US expectancies following the reminder shocks for the non-reactivated [CS2 *vs.* CS3 - stimulus x trial - $F_{1,18} = 4.24$, $P = 0.054$, $\eta_p^2 = .19$], but not the reactivated stimulus [CS1 *vs.* CS3 - stimulus x trial - $F_{1,18} < 1.17$].

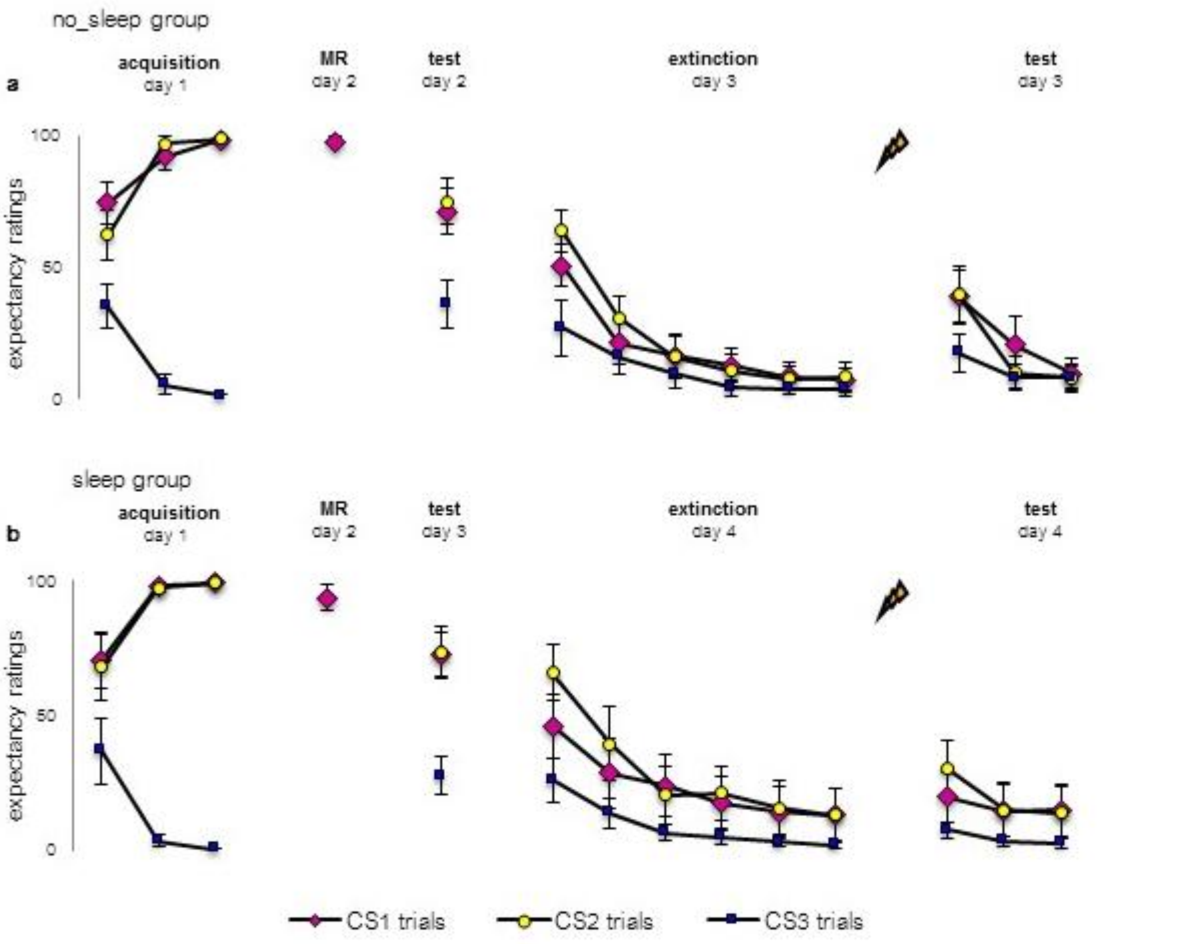

Supplementary Figure 3 | Propranolol does not affect the US expectancy ratings. Mean US expectancies to the CS1, CS2 and CS3 trials during acquisition as well as memory reactivation and test for **a.** the no_sleep group and **b.** the sleep group. MR refers to memory reactivation. Error bars represent s.e.m. US expectancy ratings did not differ between groups [i.e., simple contrasts: CS1 *vs.* CS3 and CS2 *vs.* CS3 - stimulus x trial x group - $Fs_{1,18} < 2.14$, $Ps > 0.161$]. Expectancy ratings increased during fear acquisition [CS1 *vs.* CS3 and CS2 *vs.* CS3 - stimulus x trial - $F_{1,18} = 39.82$, $P < 0.001$, $\eta_p^2 = .69$ and $F_{1,18} = 28.65$, $P < 0.001$, $\eta_p^2 = .61$] and all participants were aware of the stimulus contingencies immediately following acquisition. A drop in expectancies was observed from the last acquisition trial to the first retention test trial [CS1 *vs.* CS3 and CS2 *vs.* CS3 - stimulus x trial - $F_{1,18} = 35.23$, $P < 0.001$, $\eta_p^2 = .66$ and $F_{1,18} = 52.21$, $P < 0.001$, $\eta_p^2 = .74$], which indicates PE driven learning[1,2]. A further reduction in US expectancy ratings was observed from the first retention test trial to the last trial of extinction learning [CS1 *vs.* CS3 and CS2 *vs.* CS3 - stimulus x trial - $F_{1,18} = 13.55$, $P < 0.01$, $\eta_p^2 = .43$ and $F_{1,18} = 24.93$, $P < 0.001$, $\eta_p^2 = .58$] and in both groups we observed a return in US expectancies following the reminder shocks for the non-reactivated [CS2 *vs.* CS3 - stimulus x trial - $F_{1,18} = 4.30$, $P = 0.053$, $\eta_p^2 = .19$], but not the reactivated stimulus [CS1 *vs.* CS3 - stimulus x trial - $F_{1,18} < 2.86$, $P = 0.108$].

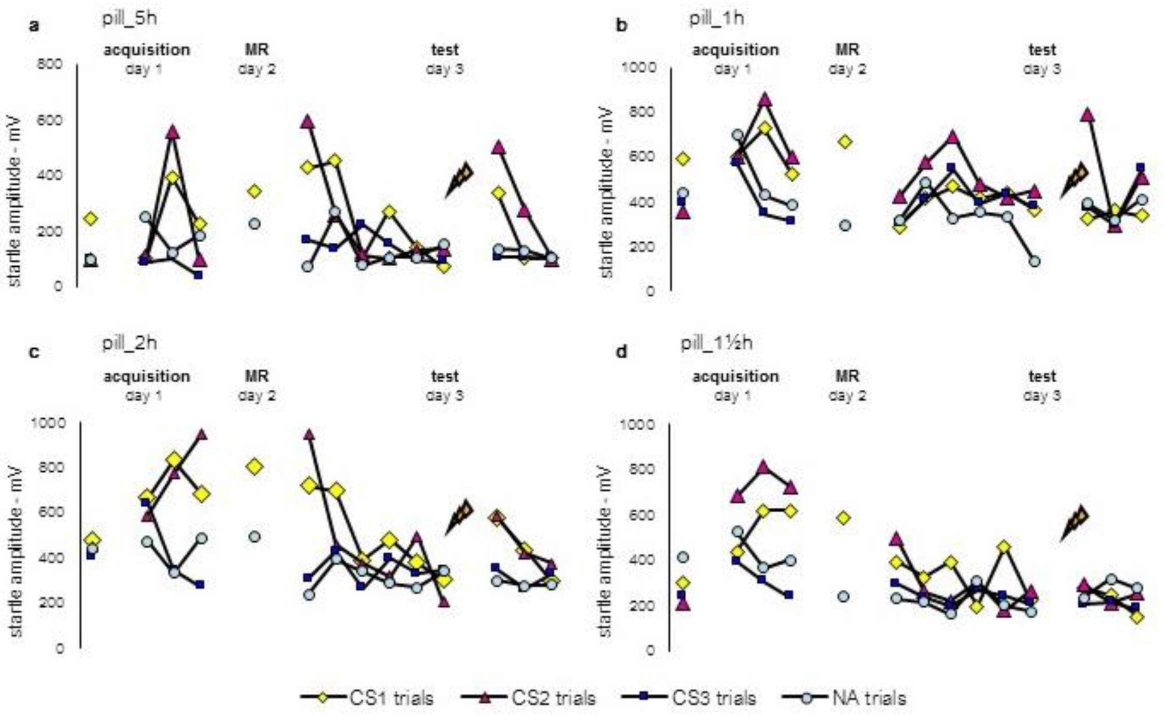

Supplementary Figure 4 | Pilots with varying timing between memory reactivation and propranolol intake. Mean startle potentiation to the fear conditioned stimuli (CS1, CS2), the control stimulus (CS3), and the noise alone (NA) trials during acquisition as well as reactivation and test. **a.** When propranolol is administered 5 h after reactivation the fear memory to the reactivated CS1 remains intact: n = 2. **b.** When the pill is administered 1 h after reactivation the fear responding to the reactivated CS1 is erased: n = 2. **c.** But when propranolol is administered 2 h after memory reactivation the fear memory to the reactivated CS1 again remains intact: n = 2. **d.** When the pill is administered 1.5 h after reactivation fear responding to the reactivation seems to remain intact as well, but the data are less clear: n = 2.

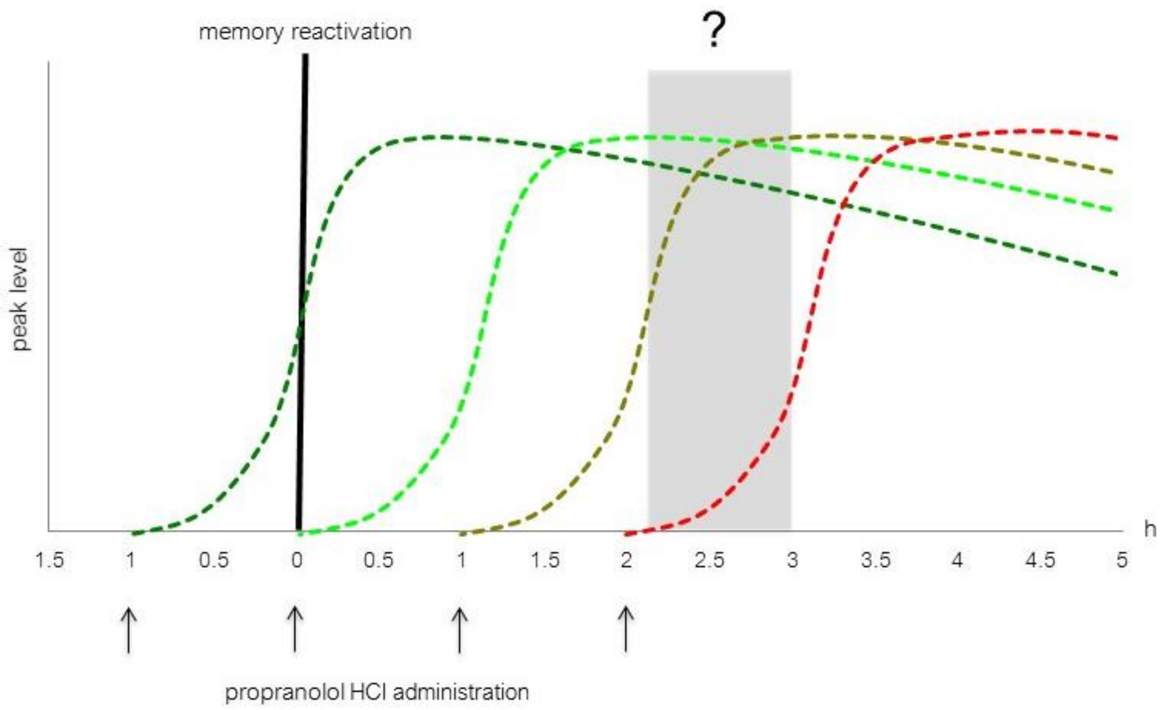

Supplementary Figure 5 | β-ARs are critical within a specific time-window. In view of the pharmacokinetics of propranolol HCl (t_{max} = 1-2 h; $t_{1/2}$ = 5 h) and the different timings of drug administration with regard to memory reactivation, we postulate a narrow and delayed time-window of β-AR activity for fear memory reconsolidation in humans. We found that drug administration either 1 h before (exp_1), directly following[1-5] or 1 h after (exp_2) memory reactivation (MR) neutralized the fear-potentiated startle response, while drug administration 2 h after MR did not affect the fear response. Together, these observations suggest that late β-AR activity 2-3 h post-reactivation is critically involved in memory reconsolidation, while β-AR activity in the first 2 h following MR is probably not decisive for memory reconsolidation.

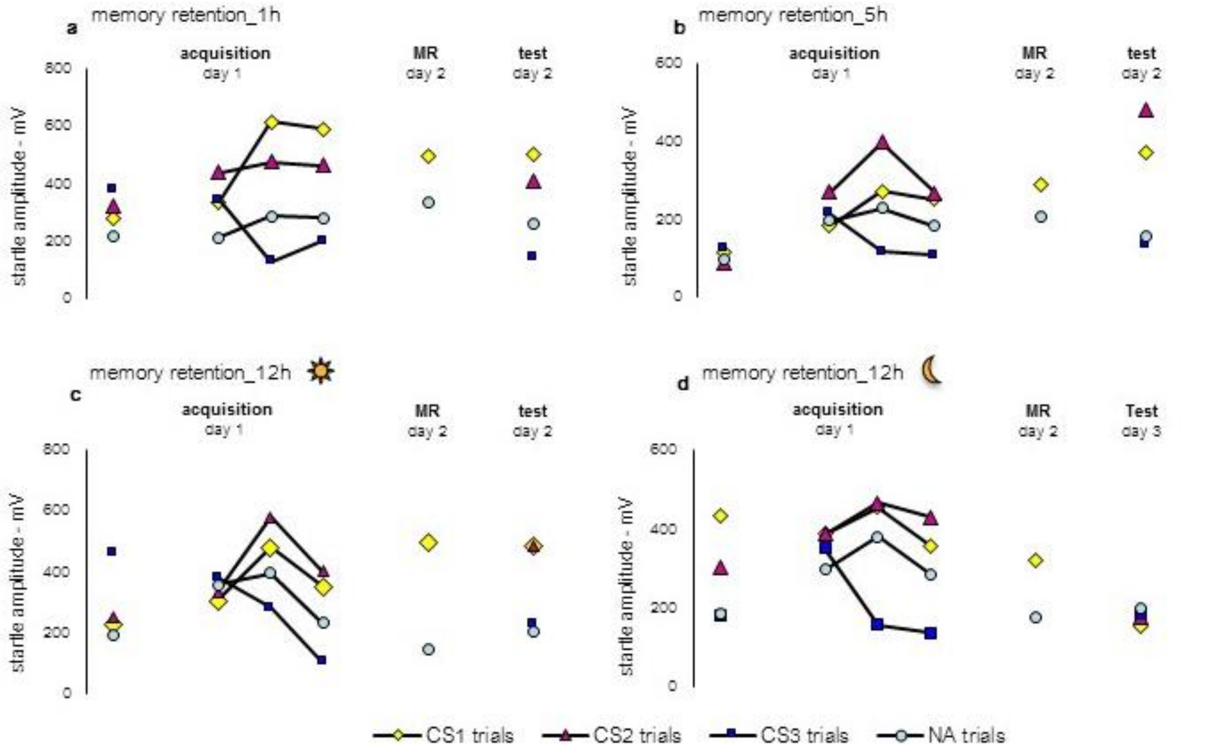

Supplementary Figure 6 | Pilots with varying timing between memory reactivation and test. Startle potentiation to the fear conditioned stimuli (CS1, CS2), the control stimulus (CS3), and the noise alone (NA) trials during acquisition as well as reactivation and test. **a.** When memory retention is tested 1 h after reactivation fear responding to the reactivated CS1 is still intact: n = 1. **b.** Inserting a memory retention test 5 h after reactivation reveals intact fear responding to the reactivated CS1 as well: n = 1. **c.** Even when memory retention was tested 12 h after reactivation the fear responding to the reactivated CS1 remained intact: n = 1. This raised the question whether sleep is necessary to observe post-reactivation amnesia. **d.** When memory retention is tested exactly 12 h after reactivation following a night of sleep the fear responding to the reactivated CS1 seems to be erased: n = 1.

Supplementary References

1. Sevenster, D., Becker, T. & Kindt, M. Prediction error governs pharmacologically induced amnesia for learned fear. *Science* **339**, 830-833 (2013).
2. Sevenster, D., Beckers, T., & Kindt, M. Prediction error demarcates the transition from retrieval, to reconsolidation, to new learning. *Learn. Mem* **21**, 580-584 (2014).
3. Soeter, M., & Kindt, M. Stimulation of the noradrenergic system during memory formation impairs extinction learning but not the disruption of reconsolidation. *Neuropsychopharmacology* **37**, 1204-1215 (2012a).
4. Soeter, M., & Kindt, M. Erasing fear for an imagined threat event. *Psychoneuroendocrinology* **37**, 1769-1779 (2012b).
5. Soeter, M., & Kindt, M. Retrieval cues that trigger reconsolidation of associative fear memory are not necessarily an exact replica of the original learning experience. *Front. Behav. Neurosci.* **9**, 122 (2015a).
6. Spielberger, C. D., Gorsuch, R. L., & Lusthene, R. E. Manual for the State-Trait Anxiety Inventory. *Consulting Psychologists Press: Palo Alto, CA* (1970).
7. Peterson, R. A., Reiss, S. Anxiety Sensitivity Index Manual. *International Diagnostic Stem: Worthington, OH* (1992).
8. Muris, P., & Merckelbach, H. A comparison of two spider fear questionnaires. *J Beh. Ther. Exp. Psychiatry* **27**, 241-244 (1996).
